# Conducting polymers take control of the field

**DOI:** 10.1073/pnas.2320855121

**Published:** 2024-01-17

**Authors:** Robert R. McLeod, George G. Malliaras

**Affiliations:** ^a^Department of Electrical, Computer and Energy Engineering and Materials Science and Engineering Program, University of Colorado, Boulder, CO 80309-0425; ^b^Electrical Engineering Division, Department of Engineering, University of Cambridge, Cambridge CB3 0FA, United Kingdom

**Fig. 1.**
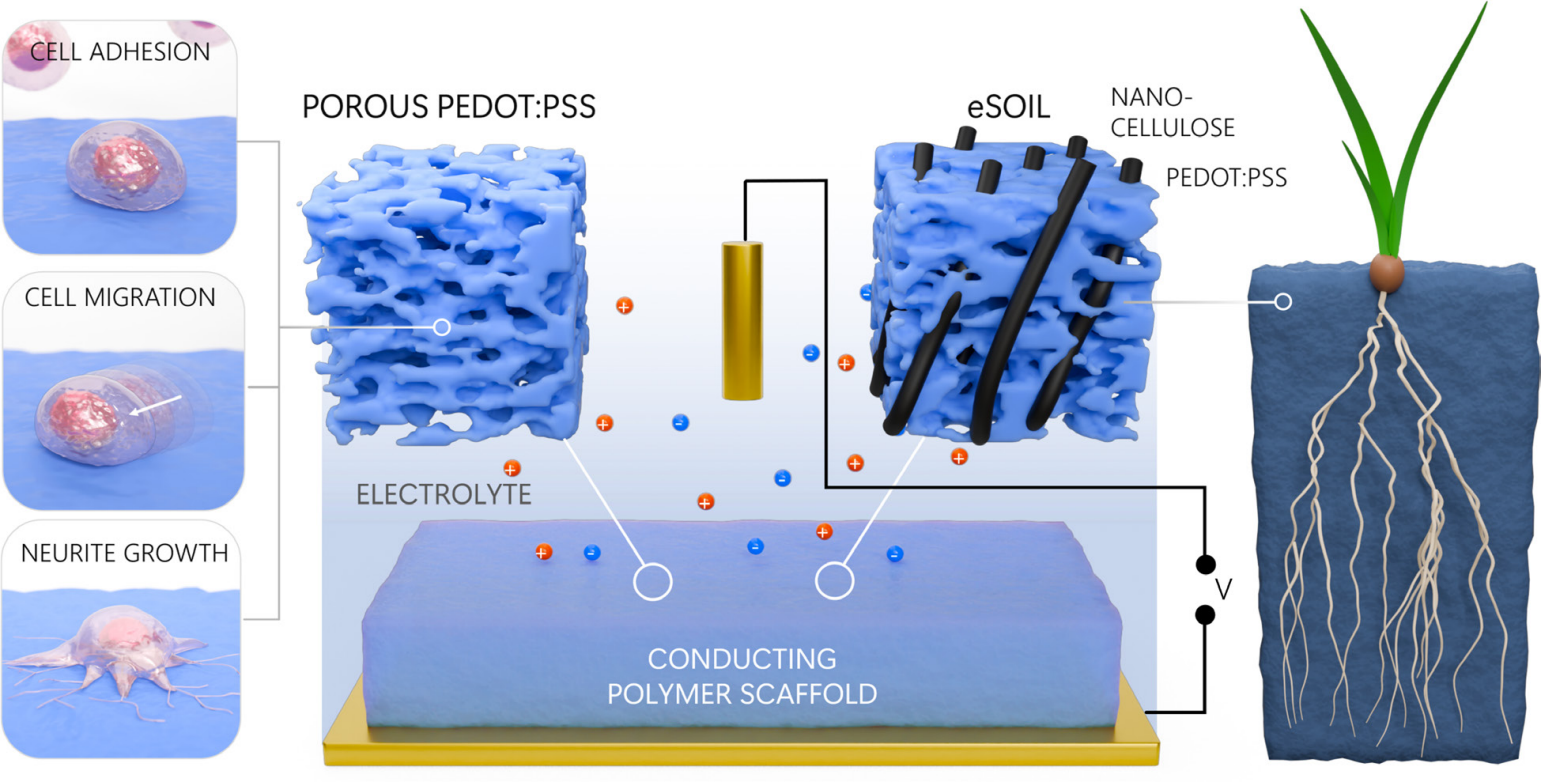
The field of organic bioelectronics has provided powerful tools for controlling cellular properties, now extended to controlling plant growth.

In PNAS, Oikonomou et al. (1) introduce the concept of eSoil, a conducting polymer–based scaffold for the hydroponic culture of plants. They show that when eSoil is polarized, it accelerates the growth of barley seedlings by 50% after 15 d of growth. This is an exciting result that may one day enable a method to increase crop yields in a sustainable fashion. Although the underlying biological processes remain to be elucidated, a key message from this work is the fact that conducting polymers enable a controlled way to study and direct the biology of plants (Fig. 1).

Conducting polymers are made by doping conjugated polymers such as polythiophenes, polyanilines, or polypyrroles. The doping process involves the oxidation or reduction of the conjugated polymer and leads to a high electronic conductivity, in the range of values that are typical for metals. A contemporary example is PEDOT:PSS, in which the polymeric semiconductor poly(3,4-ethylenedioxythiophene) (PEDOT) is doped by polystyrene sulfonate (PSS). The first observation of metal-like conductivity in a conducting polymer in the late 1970s (2) created a great deal of excitement: The idea of “synthetic metals” that are cheap to make, are lightweight, and can be processed like plastics led to a significant body of work exploring the applications of these materials in electronics and optoelectronics. This work culminated in the 2000 Nobel Prize in Chemistry.

More recently, however, the realization that conducting polymers can also support high ionic conductivity, has created a second wave of excitement for these materials (3). This combination of properties that are typical of metals (high electronic conductivity) and of electrolytes (high ionic conductivity) makes conducting polymers ideal materials for interfacing these two worlds, with applications in biology, medicine, and sustainability. One example is conducting polymer electrodes that efficiently transduce ionic currents that result from neuronal activity to map the human brain (4). Another example is conducting polymer electrodes that facilitate ion-to-electron conversion in lithium-ion batteries (5). The key parameter in these applications is a large electrochemical capacitance, arising from the volumetric interaction of ionic and electronic carriers in the conducting polymer.

At the interface with biology, conducting polymers offer additional unique characteristics that make them attractive. Their properties can be tuned via chemical synthesis including the covalent incorporation of biomolecules such as enzymes to build biosensors. They can be processed from solution and easily combined with different biopolymers to form porous 3-dimensional (3D) scaffolds that mimic the properties of their biological analogues. These scaffolds supplement the tailored biochemical and biomechanical environments of synthetic or bio-derived hydrogels with active controls including electrical stimulation via ion injection, drug release, electrochemical manipulation such as radical scavenging, and electromechanical actuation such as solvent osmosis (6). These mechanisms have been shown to control cell morphology and function (7, 8), to improve wound healing (9) and the growth of bone (10), muscle (11), and neuronal tissues (12), and to guide the development and function of stem cells (13, 14). These materials innovations are coupled with the design of new sensors and actuators that leverage mixed conductivity to deliver new diagnostic and therapeutic devices (15). The term “organic bioelectronics” has been coined to describe this burgeoning field.

Oikonomou et al. introduce the concept of eSoil, a conducting polymer–based scaffold for the hydroponic culture of plants.

As the field of organic bioelectronics is maturing, emphasis shifts beyond demonstrations of new capabilities and into detailed investigations of the biological mechanisms underlying the observed phenomena. Here is where the high electrochemical capacitance of conducting polymers can prove to be their most useful property yet as it enables one to apply an electric field in electrolytes without causing electrochemical reactions. A key limitation of most metal electrodes is the fact that they charge up, forcing the applied field to collapse at their interface with the electrolyte. To overcome this, a high voltage needs to be applied. This, however, initiates electrochemical reactions that change the composition of the electrolyte, with detrimental effects to the biological system under study. High capacitance electrodes made of conducting polymers can take a very long time to charge, during which time the applied voltage drops at the electrolyte (16). This can help avoid electrochemical reactions and pave the way for reproducible experimental conditions.

Oikonomou et al. (1) have capitalized on this property and extended the application of conducting polymer scaffolds to the plant kingdom. Their paper is an important development in the new and exciting field of “environmental bioelectronics” which applies the organic bioelectronics toolkit developed for human health to the rest of the living world. Environmental bioelectronics exploit all the advantages of conducting polymers mentioned previously as well as low-cost, solution-based manufacturing methods and combination with biodegradable materials. These materials and devices are expected to have impact as new tools for botanical science, precision agriculture, and environmental monitoring, e.g., for climate resilience. Nearly all of the biome is under study including soil, water, atmosphere, and living plants. The final category has been actively studied since initial reports demonstrated that conducting polymer circuits could be fabricated within the tissue of plants (17) and now extended to chemo-sensing of plant exudates (18), bioelectronic monitoring within living plants (19), and modulation of plant physiology (20).

Taken as a whole, these studies hint that we are at the beginning of a new era in which the natural, living world may be monitored and controlled electronically, solving critical problems in climate change and food security. Inexpensive, possibly biodegrading organic electronic sensors and actuators for living plants could provide control signals for closed-loop agriculture or drought response. Plants in hydroponic environments, as indicated by Oikonomou et al. (1), may be electronically monitored and controlled for objectives such as maturation, yield, nutrition, or disease resistance. Extrapolating further, networks of permanent biosensors throughout the biome could enable us one day to “log on” to a forest to monitor its health which in turn could be modulated by embedded bioactuators. Overall, it is clear that this field is primed for explosive growth.
